# Optimal selection of natural killer cells to kill myeloma: the role of HLA-E and NKG2A

**DOI:** 10.1007/s00262-015-1694-4

**Published:** 2015-04-29

**Authors:** Subhashis Sarkar, Michel van Gelder, Willy Noort, Yunping Xu, Kasper M. A. Rouschop, Richard Groen, Harry C. Schouten, Marcel G. J. Tilanus, Wilfred T. V. Germeraad, Anton C. M. Martens, Gerard M. J. Bos, Lotte Wieten

**Affiliations:** 1 Division of Hematology, Department of Internal Medicine, Maastricht University Medical Center+, Maastricht, The Netherlands; 2 Department of Cell Biology, University Medical Center Utrecht, Utrecht, The Netherlands; 3 Department of Transplantation Immunology, Tissue Typing Laboratory, Maastricht University Medical Center+, PO box 5800, 6202 AZ Maastricht, The Netherlands; 4 Department of Radiation Oncology (Maastro Lab), GROW School for Oncology and Developmental Biology, Maastricht University Medical Center+, Maastricht, The Netherlands; 5 Department of Immunology, University Medical Center Utrecht, Utrecht, The Netherlands; 6 Present Address: Department of Hematology, VU University Medical Center, Amsterdam, The Netherlands

**Keywords:** NK cell, Multiple myeloma, HLA, Immunotherapy, KIR, NKG2A

## Abstract

Immunotherapy with allogeneic natural killer (NK) cells offers therapeutic perspectives for multiple myeloma patients. Here, we aimed to refine NK cell therapy by evaluation of the relevance of HLA-class I and HLA-E for NK anti-myeloma reactivity. We show that HLA-class I was strongly expressed on the surface of patient-derived myeloma cells and on myeloma cell lines. HLA-E was highly expressed by primary myeloma cells but only marginally by cell lines. HLA-E^low^ expression on U266 cells observed in vitro was strongly upregulated after in vivo (bone marrow) growth in RAG-2^−/−^ *γ*c^−/−^ mice, suggesting that in vitro HLA-E levels poorly predict the in vivo situation. Concurrent analysis of inhibitory receptors (KIR2DL1, KIR2DL2/3, KIR3DL1 and NKG2A) and NK cell degranulation upon co-culture with myeloma cells revealed that KIR–ligand-mismatched NK cells degranulate more than matched subsets and that HLA-E abrogates degranulation of NKG2A+ subsets. Inhibition by HLA-class I and HLA-E was also observed with IL-2-activated NK cells and at low oxygen levels (0.6 %) mimicking hypoxic bone marrow niches where myeloma cells preferentially reside. Our study demonstrates that NKG2A-negative, KIR–ligand-mismatched NK cells are the most potent subset for clinical application. We envision that infusion of high numbers of this subclass will enhance clinical efficacy.

**Electronic supplementary material:**

The online version of this article (doi:10.1007/s00262-015-1694-4) contains supplementary material, which is available to authorized users.

## Introduction

Multiple myeloma is caused by expansion of malignant plasma cells in the bone marrow (BM) and remains largely incurable with the current treatment options [1]. Allogeneic hematopoietic stem cell transplantation (allo-HSCT) performed over HLA-C barriers can provide curative anti-myeloma responses [2]. However, poor clinical outcomes and T cell-mediated graft-versus-host disease (GvHD) are major limitations of allo-HSCT for myeloma [3–6]. Infusion of alloreactive natural killer cells (NK) cells could be an appealing strategy to improve clinical effectiveness since the risk of GvHD is low and there is no need for myeloablative conditioning [7, 8].

NK cells are large granular lymphocytes with the potential to selectively kill malignant cells. The balance between tolerance to healthy cells and eradication of virally infected or tumor cells is established by a panel of activating and inhibitory receptors on the NK cell surface [9]. Activating receptors, interacting with “stress-induced flags” on diseased cells, trigger NK cell killing. Inhibitory receptors bind to self-molecules such as HLA-class I and HLA-E that are expressed by virtually every normal cell and protect against unwanted NK cell reactivity. An important class of inhibitory receptors, the killer immunoglobulin-like receptors (KIRs), interacts with HLA-class I molecules on the target cell; KIR2DL1 ligates HLA-C group 2 (Lsy80) alleles, KIR2DL2/3 ligates HLA-C group 1 (Asn80) alleles and KIR3DL1 interacts with HLA-Bw4 motifs [10, 11]. The lectin-like family member NKG2A interacts with HLA-E [12].

Upon HLA-mismatched allo-HSCT, donor NK cells eliminate tumor cells when the patient lacks HLA epitopes from the donor, and donor NK cells express KIRs specific for these epitopes (KIR-ligand mismatch) [13]. Landmark studies in the haplo-identical HSCT setting showed that KIR–ligand-mismatched NK cells exert anti-leukemic effects and improve survival of acute myeloid leukemia (AML) patients [14, 15]. Also in myeloma, incompatibility between donor KIRs and patient HLA has been associated with a better clinical outcome [2]. Moreover, a first clinical trial showed that isolated KIR–ligand-mismatched NK cells can be safely administrated to myeloma patients though the contribution of NK cells, in addition to the conditioning regime, could not be established in this trial [16]. Beneficial effects of KIR–ligand-mismatched NK cell infusion have been observed in other types of cancer as well [17–20]. Although these studies are encouraging, optimization of the clinical efficacy of transferred NK cells is still required.

While there are several reasons why NK cells therapy could be a major step forward in immunotherapy of cancer, various mechanisms have been described that could reduce clinical effectiveness, e.g., decreased expression of activating receptors [21–23], hypoxia [24], the expression of immunosuppressive soluble or surface molecules [25–27] or infusion of insufficient numbers of (the right) NK cells and HLA-class I expression by myeloma cells [28–30]. Genotypic selection of a KIR–ligand-mismatched donor seems to be a good strategy to reduce inhibitory effects by HLA-class I. However, because NK cells can express one or a combination of inhibitory receptors, not all donor NK cells will exclusively express KIRs that are mismatched with HLA-class I of the patient. Furthermore, of all peripheral blood NK cells, 20–80 % expresses NKG2A [31, 32]. For clinical-grade expanded NK cells, from either hematopoietic progenitor cells [33], blood-derived NK cells [34], or pluripotent stem cells [35], this percentage can be higher than 80 %. Because HLA-E is low polymorphic and all functional HLA-E alleles can inhibit NK cells through NKG2A [36], NKG2A could abolish the overall NK cell response against HLA-E-expressing tumors, even in the KIR–ligand-mismatched setting. This makes HLA-E clinically highly relevant, but information on HLA-E expression and its inhibitory potential in myeloma is lacking.

In the present study, aimed to refine the selection process for NK cell therapy, we studied the relevance of HLA-class I and HLA-E for anti-myeloma reactivity of allogeneic NK cells. First, we determined the presence of HLA-class I and HLA-E on primary myeloma cells and on myeloma cell lines, revealing that primary myeloma cells express both HLA-class I and HLA-E. Also, we found major differences with myeloma cells cultured in vitro and the same cells grown in vivo. Second, we assessed the inhibitory potential of HLA-class I and HLA-E on different NK cell subsets in a co-culture system that allowed synchronized analysis of degranulation of individual NK cells subsets, demonstrating the potent inhibitory effects of HLA-class I and HLA-E on NK cell alloreactivity. To better predict the in vivo response, we performed these assays at ambient and clinically relevant low oxygen levels.

## Materials and methods

### Cell culture incubators

Experiments were performed in ambient 95 % humidified air, 21 % O_2_ incubator (SANYO) containing 5 % CO_2_. Hypoxia experiments were completely performed inside 0.6 % O_2_ incubators (Invivo_2_, 1000 Ruskinn Technology Ltd) at 37 °C with 5 % CO_2_.

### Cell lines

Myeloma cell lines [37] and HLA-class I-deficient K562 cell line (ATCC) were cultured in RPMI-1640 medium (Gibco) supplemented with 10 % fetal calf serum (Integro), 1000 U/mL penicillin (Gibco) and 100 µg/mL streptomycin (Gibco). The LME-1 cell line (received through Dr. R. van Oers, AMC, Amsterdam) was cultured in IMDM medium (Gibco), and XG-1 in IMDM medium with (500 pg/ml) IL-6 (Biosource).

### Flow cytometric analysis of HLA-E and HLA-class I expression

Material from myeloma and plasma cell leukemia patients was obtained as a waste product from a diagnostic procedure that had been approved by the hospital’s medical ethical committee. Bone marrow cells were stained for CD38 (HB7, BD), CD138 (CLB-1D4, Sanquin), cytoplasmic kappa light chain (G20-193, BD), cytoplasmic lambda light chain (1–555–2, BD), HLA-class I (G46-2.6, BD) or HLA-E (3D12, eBioscience). HLA expression was determined on CD38^high^ cells. All flow cytometry-based assays were performed on a BD FACS Canto II. Data were analyzed with BD FACSDiva v6.1.2 or FlowJo 7.6.

### HLA genotyping and NK cell donor selection

Genotypic expression of HLA epitopes (HLA-C1, HLA-C2 and HLA-Bw4) in cell lines (supplemental table S1) and in healthy volunteers was determined by sequence-specific oligonucleotides (SSO) analysis and Luminex^®^ according to the manufacturer’s guidelines (One Lambda). Healthy volunteers, with genotype HLA-C1+C2+Bw4+ and phenotypically expressing KIR2DL1, KIR2DL2/3 and KIR3DL1 were selected as NK cell donors. Donors signed informed consent forms.

### NK cell isolation and CD107a degranulation assay

NK cells were isolated from blood by negative MACS selection (Miltenyi Biotec) according to manufacture's instructions. NK cells were activated with 1000 U/ml IL-2 (Proleukin) for 6 h at 21 % O_2_. Activated NK cells were co-cultured with target cells at 1:1 ratio in 96-well round-bottom plates in duplicate with anti-CD107a-Horizon-V450 (H4A3, BD). After 1 h, co-culture at 21 % O_2_ or 0.6 % O_2_, monensin (BD GolgiStop, Cat# 554724) was added. After another 8–9 h, co-cultures were stained on ice with anti-CD3-APC/H7 (SK7, BD), anti-CD56-PeCy7 (B159, BD), anti-KIR2DL1-APC (143211, R&D), anti-KIR2DL2/3/S2-PE (DX27, Miltenyi Biotec), anti-KIR3DL1-FITC (DX9, Miltenyi Biotec) and anti-NKG2A-PC5.5 (Z199, Beckman Coulter). For co-cultures with IL-2-activated NK cells, target cells were pre-incubated for 6 h at 21 % O_2_ or 0.6 % O_2_. For experiments without IL-2 activation, target cells cultured at 21 % O_2_ were used. Target cells were subsequently co-cultured with freshly isolated NK cells in a 10- to 12-h degranulation assay. For HLA-E blocking experiments, target cells were pre-incubated for 30 min at 37 °C with 10 µg/ml of anti-HLA-E (3D12; IgG1 isotype eBioscience) or IgG1 isotype control. Gating strategy is described in supplemental figure S2.

### HLA-E induction by peptides

U266 cells were incubated with 500 µM of HLA-A1 (VMAPRTLLL), HLA-B7 (VMAPRTVLL) or a control peptide (RGPGRAFVTI) for 12 h at 37 °C. As additional negative controls, U266 cells were incubated without peptide or with DMSO, the solvent of the peptides. After 12 h, HLA-E expression was determined by flow cytometry. For degranulation assays, peptide incubation was done for 2 h after which the cells were co-cultured with IL-2-activated NK cells in a 12-h degranulation assay.

### Mice and multiple myeloma disseminated growth model in RAG-2^−/−^*γ*c^−/−^ mice

RAG-2^−/−^ *γ*c^−/−^ mice were bred in the SPF unit Central Animal Facility of the Utrecht University using filter top cages. After a total body irradiation (3.0 Gy 200 kV X-rays), the mice received an injection with 5 × 10^6^ cells intravenously from the green fluorescence protein (GFP)–luciferase-marked, human MM cell line U266. Tumor growth of the U266-luc cell line was monitored by bioluminescence imaging as described previously [37]. Upon killing the mice, BM cells were stained for human-CD45, HLA-class I and HLA-E.

### Statistical analyses

Statistical significances of differences were determined by paired *t* test or two-way repeated measures ANOVA with Bonferroni correction. A *p* value of <0.05 was considered significant. Analysis was performed with GraphPad Prism V (Graphpad Software Inc).

## Results

### Primary myeloma cells express HLA-class I and HLA-E on the cell surface

**Fig. 1:**
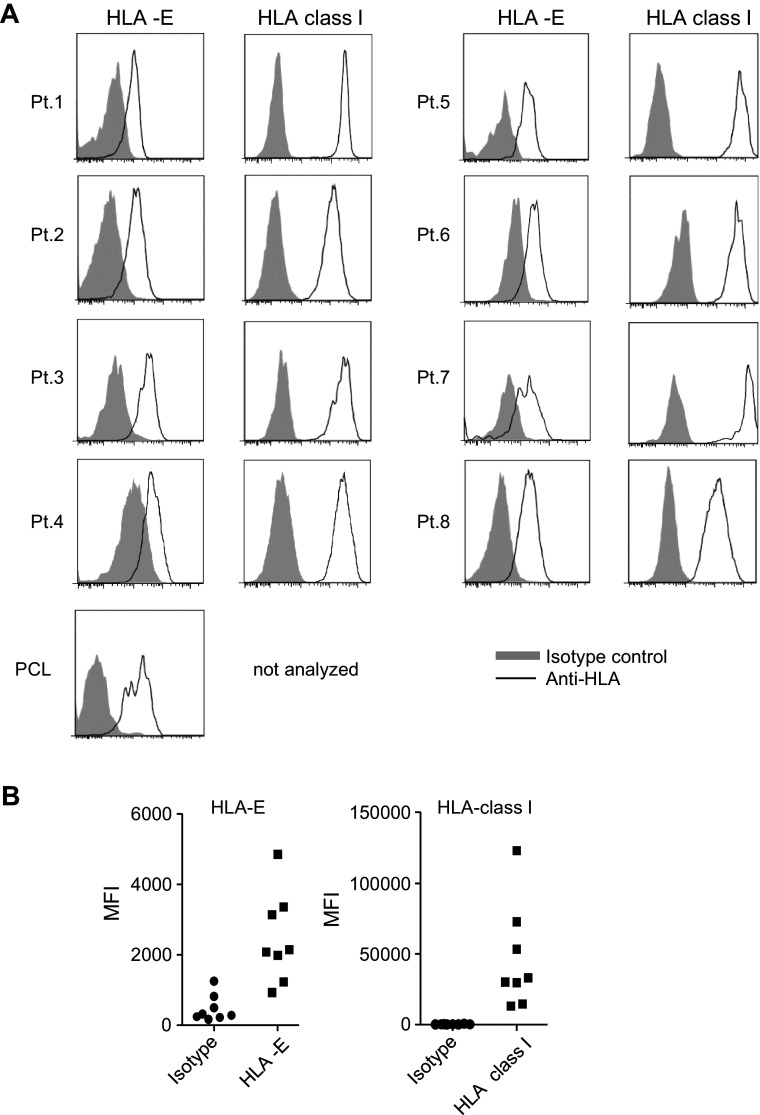
Patient-derived primary myeloma cells express HLA-class I and HLA-E on the cell surface. Mononuclear cells obtained from bone marrow aspirates of patients with myeloma (*n* = 8) or plasma cell leukemia (PCL; *n* = 1) were stained for CD38, HLA-class I and HLA-E and were analyzed by flow cytometry. **a** Histograms show HLA-class I or HLA-E cell surface expression (*open* histograms) on CD38^high^ cells for the myeloma or PCL patients. Matched isotype controls are depicted in *gray* histograms. **b** Graph shows MFI data obtained from the histograms. *Each dot* depicts data from one patient

To study HLA expression on primary myeloma cells, cells were obtained from BM aspirates of eight myeloma patients and one plasma cell leukemia (PCL) patient. Directly upon isolation, surface expression of HLA-class I and HLA-E was analyzed by flow cytometry. Plasma cells were identified as CD38^high^ and displayed skewed intracellular expression of either kappa or a lambda light chain indicative for myeloma (supplemental figure S1). In all myeloma patients, CD38^high^ cells were positive for HLA-E and HLA-class I (Fig. 1). Also, CD38^high^ cells from the PCL patient expressed HLA-E. The level of HLA-E and HLA-class I on CD38^high^ cells was comparable to the level observed on normal BM cells of the same patient or on plasma cells from a non-myeloma patient (data not shown).

### Myeloma cell lines express high levels of HLA-class I and heterogeneous levels of HLA-E

**Fig. 2:**
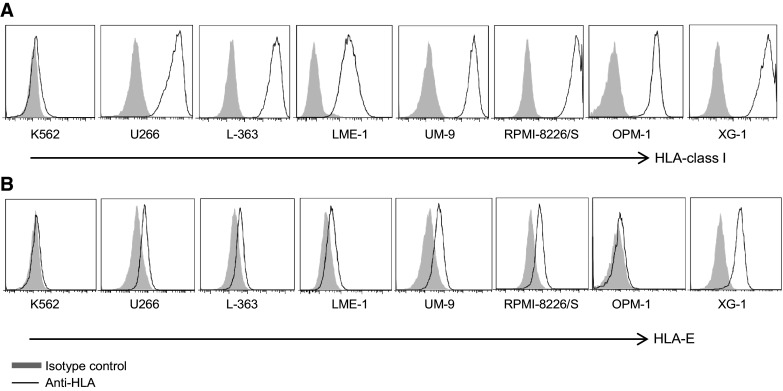
Myeloma cell lines express high levels of HLA-class I and heterogeneous levels of HLA-E. HLA-class I **a** and HLA-E **b** surface expression of HLA-class I-deficient K562, and seven myeloma cell lines (U266, L-363, LME-1, UM-9, RPMI-8226/S, OPM-1, XG-1) was determined by flow cytometry. Histograms are representative of three different measurements. HLA expression is depicted by open histograms and matched isotype controls by *gray* histograms

Surface expression of HLA-class I and HLA-E was also assessed on a panel of myeloma cell lines including U266, L-363, LME-1, UM-9, RPMI-8226/S, OPM-1 and XG-1, and on the leukemia cell line K562. This revealed that all myeloma cell lines strongly expressed HLA-class I (Fig. 2a). K562 cells were almost completely negative for HLA-class I. The cell lines differed in expression levels of HLA-E; K562 and OPM-1 lacked cell surface HLA-E, while U266, L-363, UM-9, LME-1 and RPMI-8226/S expressed low levels of HLA-E (<1 log difference with the isotype control). XG-1 expressed intermediate HLA-E levels (approximately 1 log difference with the isotype control) (Fig. 2b).

### In vivo grown U266 myeloma cells express higher levels of HLA-E than in vitro grown U266 cells

**Fig. 3:**
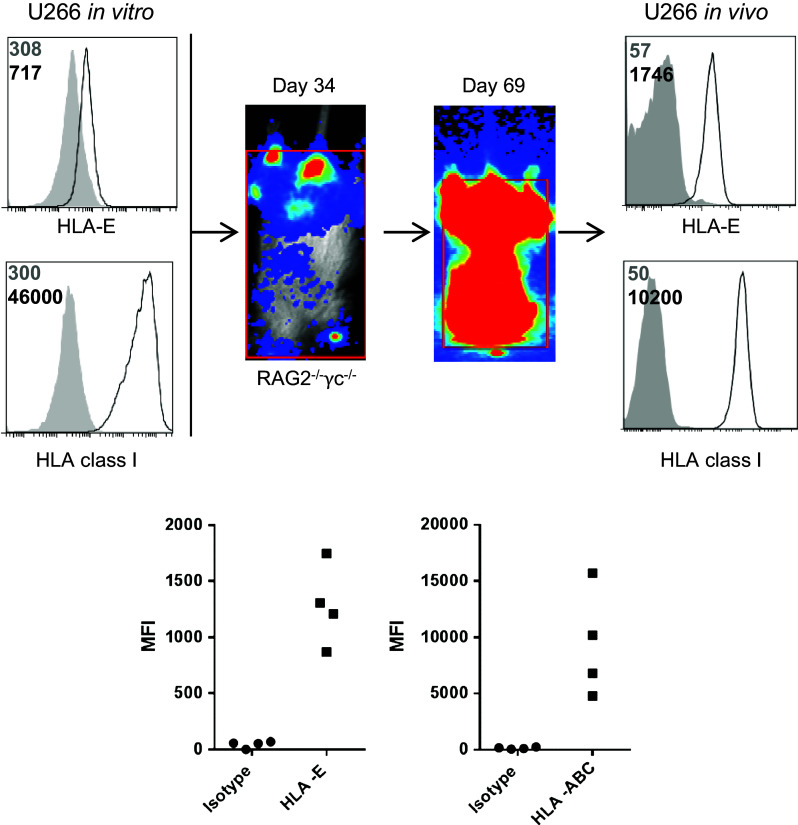
In vivo grown U266 myeloma cells have a higher HLA-E expression than in vitro cultured U266 cells. 5*10^6^ U266 cells were injected in RAG-2^−/−^ *γ*c^−/−^ immunodeficient mice. Tumor growth was monitored at multiple time points after injection by bioluminescent imaging. Once the human endpoint was reached, mice were killed and the bone marrow was harvested and stained for HLA-class I and HLA–E. U266 cells grown in vitro were also stained for HLA-class I and HLA-E. HLA expression was analyzed by flow cytometry. Representative histograms are shown of HLA expression (*open* histograms) and matched isotype controls (*gray* histograms). Numbers in the histograms depict MFI of the isotype control (*gray*) or HLA (*black*). Graphs show MFIs obtained from histograms of in vivo HLA expression. *Each dot* represents one mouse

As we observed a clear expression of HLA-E on all patient-derived CD38^high^ cells, but only low expression on in vitro cultured myeloma cell lines, we compared HLA-E expression on in vitro grown U266 cells with U266 cells after in vivo passaging. To this end, GFP–luciferase-marked U266 cells were injected in RAG-2^−/−^ *γ*c^−/^mice, thereby providing the cells with their natural BM environment. Tumor growth was monitored with bioluminescence imaging. At end-stage myeloma development, the BM was harvested and tumor cells, identified by GFP and human leukocyte marker CD45, were analyzed for surface HLA-E and HLA-class I. This analysis revealed that both in vitro and in vivo grown U266 cells strongly expressed HLA-class I, albeit that the in vivo level was somewhat lower than the in vitro level. A striking observation was that the in vivo passaged U266 cells expressed much higher levels of HLA-E when compared to U266 cells grown in vitro (Fig. 3).

### KIR–ligand-mismatched NK cell subsets mediate the most effective anti-myeloma response

To evaluate the functional relevance of HLA for NK cell anti-myeloma alloreactivity, myeloma cell lines were co-cultured with NK cells from donors expressing all three inhibitory epitopes (i.e., HLA-C1+, HLA-C2+ and HLA-Bw4+). To enable comparative analysis of anti-myeloma activity of NK cell subsets, cells were stained for KIRs and NKG2A, and NK cell degranulation of subsets was assessed by flow cytometric analysis for the degranulation marker CD107a (supplemental figure S2). Previously, we and others showed that CD107a is a reliable surrogate marker for NK cell cytotoxicity [24, 38] and traditional cytotoxicity assays as such would not allow the analysis of subgroups without cell sorting.

**Fig. 4:**
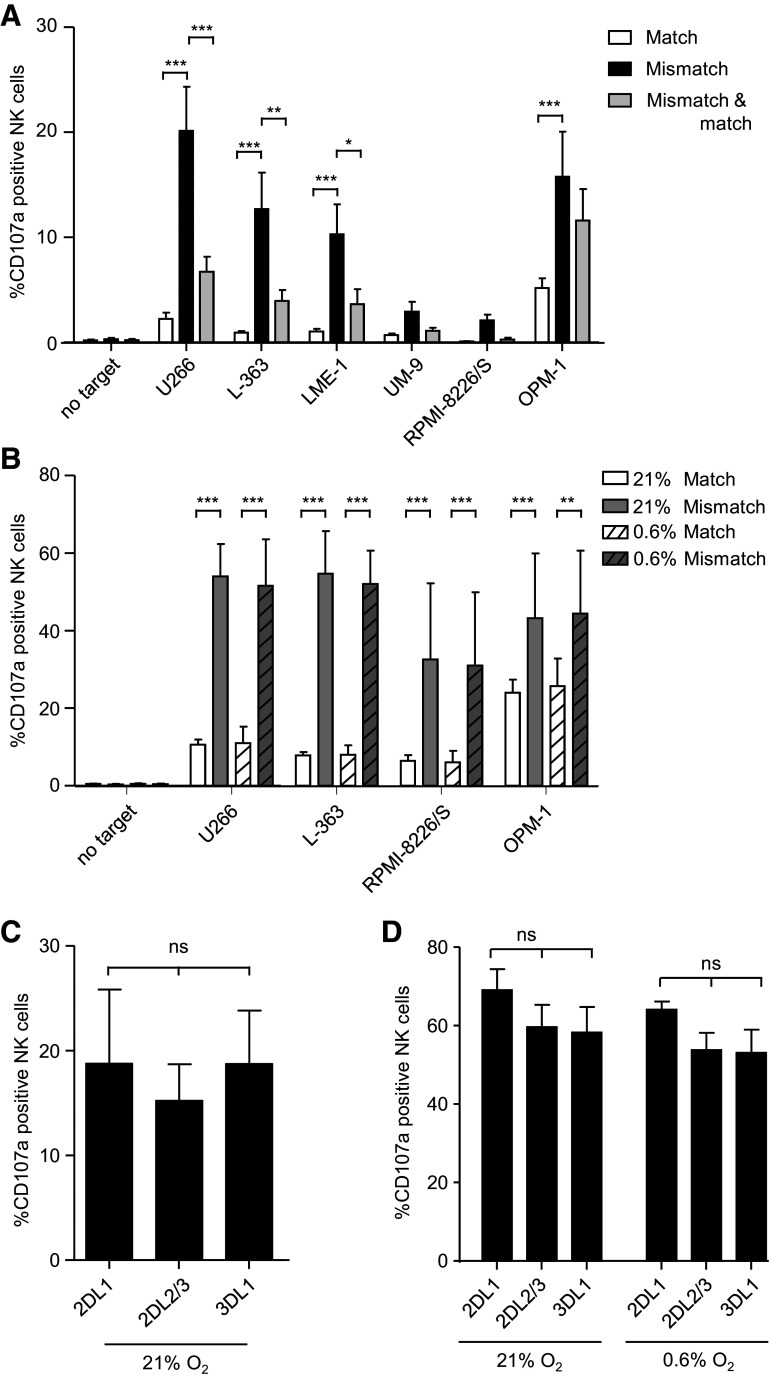
KIR–ligand-mismatched NK cell subsets mediate the most effective anti-myeloma response. **a** Six myeloma cell lines were co-cultured with NK cells isolated from peripheral blood in the presence of anti-CD107a at 21 % of oxygen. After 12 h, cells were stained for KIRs and NKG2A and degranulation (CD107a+) was measured by flow cytometry. For analysis, NK cells (CD3-CD56+) were subdivided into 16 subpopulations based on their expression of different inhibitory receptors and the percentage of CD107a+ cells per subset was analyzed. Next, the 16 populations were categorized into three subgroups: KIR–ligand-matched NK cells (*white bars*), KIR–ligand-mismatched NK cells (*black bars*) and NK cells with a co-expression of KIR–ligand-mismatched and matched cells (*gray bars*). This was done per myeloma cell line based on the HLA genotype of the cell line. For all subsets, NKG2A+ NK cells were excluded from the analysis. Graph shows mean percentage and SEM of CD107a+ NK cells for each of these subgroups. **b** Myeloma cell lines were pre-incubated for 6 h at 21 % or 0.6 % O_2_. NK cells were activated for 6 h with 1000 U/ml IL-2 at 21 % of oxygen. After 6 h, NK cells and myeloma cells were co-cultured for an additional 10 h at 21 % or 0.6 % O_2_. Analysis of degranulation of IL-2-activated NK cells subsets was performed as described under **a**. Graph shows mean percentage and SEM of CD107a+ NK cells, for NK cells exclusively expressing matched (*white bars*) or mismatched KIRs (*gray bars*) at 21 % O_2_ (*open bars*) or 0.6 % O_2_ (*dashed bars*). Unactivated NK cells (**c**) or activated NK cells (**d**) were co-cultured with K562 as described in **a** and **b**. Graphs in **c** and **d** show mean percentage and SEM of CD107a+ NK cells for subsets exclusively expressing KIR2DL1 or KIR2DL2/3 or KIR3DL1. Experiments in **a** and **c** were performed in the same assay in duplicate cultures for *n* = 4 healthy donors. Experiments in **b** and **d** were performed in the same assay in duplicate cultures for *n* = 5 donors. *p < 0.05, **p < 0.01, ***p < 0.001

To assess the importance of KIR–HLA class I–KIR interaction, NKG2A−KIR+ NK cells were classified into three subsets based on the target HLA-class I genotype (supplemental table S1): a subset exclusively expressing matched KIRs, a subset expressing only mismatched KIRs, and a subset co-expressing matched and mismatched KIRs. For all three subsets, the percentage of CD107a+ cells was below 1 % without target cells (Fig. 4a). The percentage of degranulating CD107a+ matched NK cells upon culture with myeloma cell lines was also low, 5.1 % with OPM-1 and <2.2 % with the other cell lines. For the mismatched subsets, the percentage of degranulating NK cells was higher than the percentage of the matched subset. The magnitude differed between the target cell lines: 20.1 % with U266, 12.7 % with L-363, 10.3 % with LME-1, 2.9 % with UM-9, 2.1 % with RPMI-8226/S and 15.8 % with OPM-1. For the NK cell subset co-expressing both matched and mismatched KIRs, percentages of CD107a+ cells were in between that of the subset exclusively expressing matched KIR and the subset exclusively expressing mismatched KIR. Together, these data illustrated that anti-myeloma NK cell reactivity is abrogated by HLA-class I.

Since clinical NK cell products usually consist of cytokine-activated or cytokine-expanded NK cells [16, 39, 40], we investigated whether our observation that unactivated KIR–ligand-mismatched NK cells are better responders to myeloma than matched NK cells was also true for activated NK cells. To test this, NK cells were activated for 6 h with IL-2 prior to the analysis of degranulation of the different NK cell subsets. Spontaneous degranulation of activated NK cells was again low (<0.5 %). For matched NK cells, the percentage of CD107a+ cells in response to myeloma target cells was 10.6 % with U266, 7.8 % with L-363, 6.4 % with RPMI-8226/S and 24 % with OPM-1 (Fig. 4b). In line with our first observation, NK cells that were KIR–ligand mismatched showed a significantly better anti-MM response than the matched subsets; the percentages of CD107a+ cells were 54 % with U266, 54.8 % with L-363, 32.6 % with RPMI-8226/S and 43.2 % with OPM-1.

Previously, we showed that hypoxia can inhibit the cytotoxic capacity of NK cells and that IL-2 activation of NK cells can compensate for that [24]. As the myeloma microenvironment exhibits hypoxic regions [41], we also determined degranulation of IL-2-activated NK cell subsets at physiologically relevant hypoxic culture conditions of 0.6 % O_2_. In response to all tested cell lines and for both matched and mismatched subsets, the percentage of CD107a+ cells was comparable to the levels observed at 21 % of oxygen. Thus, also under hypoxic culture conditions, KIR–ligand-mismatched subsets displayed superior anti-myeloma activity (Fig. 4b).

As a control, degranulation of KIR2DL1, KIR2DL2/3 or KIR3DL1 single positive NK cells in response to the HLA-class I negative K562 cell line was determined. For the unactivated NK cells, this showed that all three subsets had around 18–20 % CD107a+ NK cells upon incubation with K562 (Fig. 4c). Also for the activated NK cells, there was no significant difference in the percentage of CD107a+ cells between KIR2DL1, KIR2DL2/3 and KIR3DL1 single positive subsets, either at 21 % O_2_ or at 0.6 % O_2_ (Fig. 4d), demonstrating that all subsets can degranulate to a comparable extent in the absence of inhibitory signaling by KIR-ligand interaction.

### HLA-E can abrogate the anti-myeloma response of NKG2A+ NK cells

**Fig. 5:**
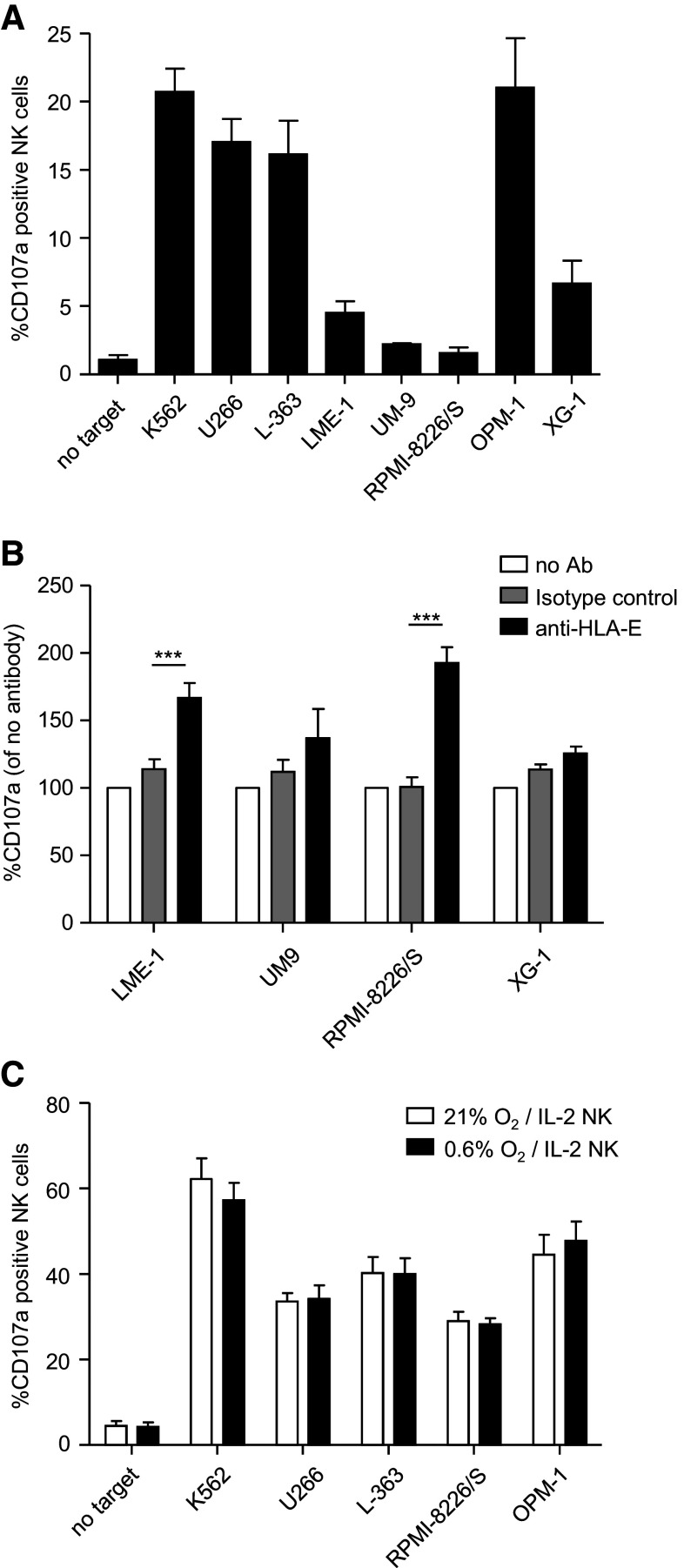
HLA-E can abrogate the anti-myeloma response of NKG2A+ NK cells. **a** The tumor cell lines were co-cultured at 21 % O_2_ with freshly isolated peripheral blood-derived NK cells for 12 h, in the same assay as in Fig. 4 **a**/**c**. NK cells expressing only NKG2A as an inhibitory receptor were analyzed downstream for CD107a expression. Graph shows mean percentage and SEM CD107a+ of NKG2A+ KIR− NK cells. **b** LME-1, UM-9, RPMI-8226/S and XG-1 were co-cultured with non-activated NK cells for 12 h at 21 % O_2_, in the presence of an HLA-E-blocking antibody (*black bars*), an IgG1 isotype control (*gray bars*) or without antibody (*white bars*). Graph shows mean normalized percentage of CD107a+ cells of NKG2A+ KIR− NK cells (% with Ab divided by % without antibody).** c** Myeloma cell lines were pre-incubated for 6 h at 21 % or 0.6 % O_2_. NK cells were activated with 1000 U/ml IL-2 at 21 % O_2_. Thereafter, they were co-cultured with K562, U266, L363, RPMI-8226/S and OPM-1 for 10 h at 21 % O_2_ or at 0.6 % O_2_, and CD107a expression on KIR-NKG2A+ NK cells was analyzed as described in Fig. 4. Graph shows mean percentage and SEM CD107a+ of NKG2A+ KIR− NK cells. All experiments were performed in duplicate and with *n* = 5 donors. ***p < 0.001

To evaluate the functional relevance of HLA-E, we used the experimental setup described above and determined degranulation of NK cells expressing only the inhibitory receptor for HLA-E, i.e., NKG2A. In the absence of target cells, 1 % of KIR-NKG2A+ NK cells was positive for CD107a (Fig. 5a). Between 16 and 21 % of KIR-NKG2A+ NK cells expressed CD107a upon co-culture with U266, L-363 and OPM-1. This percentage was only slightly lower than the percentage observed with the HLA-negative K562, suggesting that the low levels of HLA-E on U266, L-363 and OPM-1 were not sufficient to inhibit the KIR-NKG2A+ NK cells. In response to LME-1, UM-9, RPMI-8226/S and XG-1, only 2–6 % of the KIR-NKG2A+ NK cells was positive for CD107a. To investigate whether the poor response against these targets was the consequence of HLA-E, despite its low expression (Fig. 2), HLA-E was blocked on these four cell lines with an anti-HLA-E antibody. For UM-9 or XG-1, blocking did not significantly increase NK cell degranulation as compared to the isotype control (Fig. 5b). However, for LME-1 or RPMI8226/S, blocking enhanced degranulation of KIR-NKG2A+ NK subsets of all four donors. This enhanced degranulation was not observed in NKG2A−KIR+ subsets (data not shown), suggesting that the effect was HLA-E specific. Nevertheless, we cannot completely exclude the possibility that the HLA-E antibody had triggered antibody-mediated cellular cytotoxicity by binding to Fc receptors. Hence, although HLA-E is not the only contributing factor, our data show that HLA-E can clearly inhibit the anti-myeloma response of KIR-NKG2A+ NK cells.

We also studied degranulation of IL-2-activated KIR-NKG2A+ NK cells upon co-culture with K562, U266, L-363, RPMI-8226/S and OPM-1. Spontaneous degranulation without target cells was 5 %. In response to K562, 60 % of the KIR-NKG2A+ NK cells expressed CD107a, while this was 30–50 % in response to the myeloma cells (Fig. 5c). This phenomenon was also valid for targeting myeloma in a hypoxic environment of 0.6 % oxygen, showing that degranulation of activated NK cells was not severely hampered by the low levels of HLA-E present on the myeloma cell lines.

### Upregulation of HLA-E on myeloma cells diminishes degranulation of IL-2-activated NKG2A+ NK cells

**Fig. 6:**
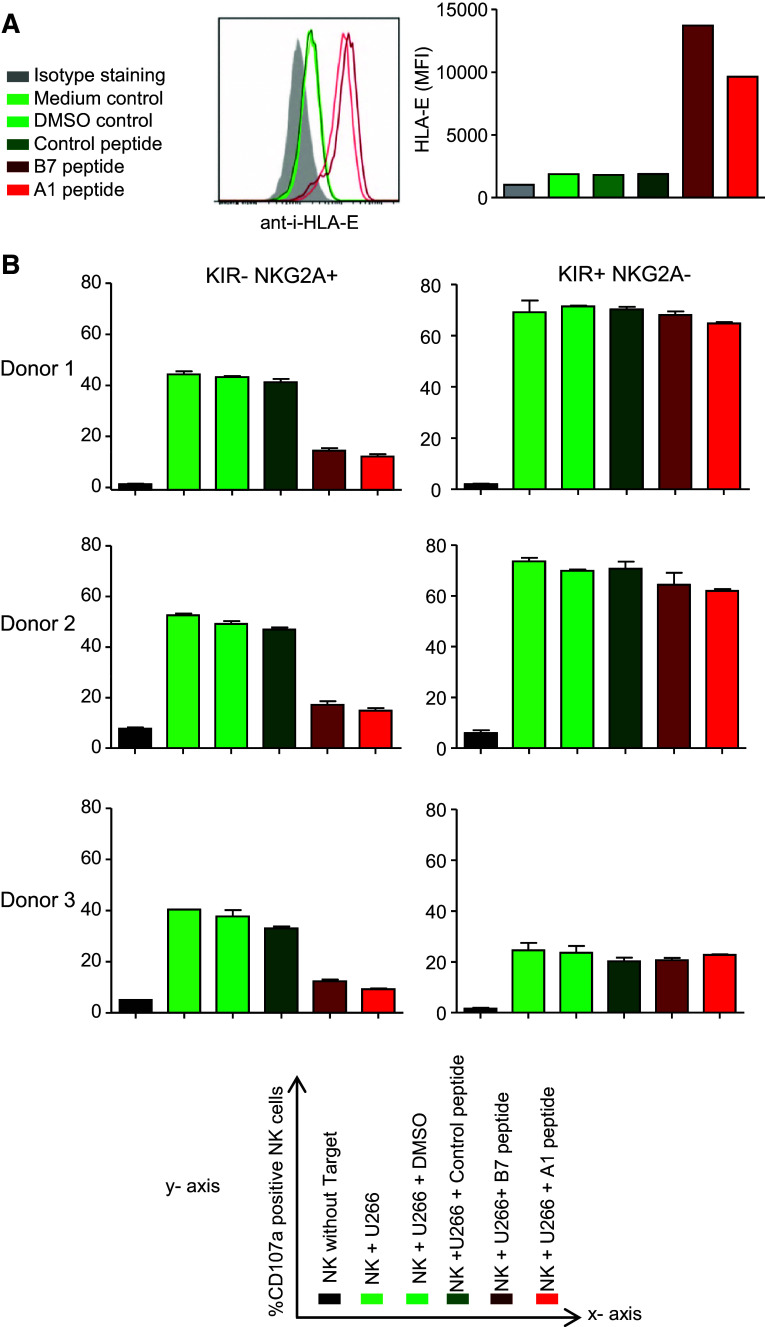
Upregulation of HLA-E renders NK-sensitive myeloma cells resistant to IL-2-activated NKG2A+ NK cells. **a** U266 cells were incubated for 12 h at 37 °C with 500 µM A1- or B7- HLA-E binding peptides. As control, U266 cells were incubated with a control (non-binding) peptide, DMSO or medium. HLA-E surface expression was determined by flow cytometry after staining of the cells with anti-HLA-E or an IgG1 isotype control. Shown is a representative histogram with, in *gray*, the isotype control staining, in *green*, the control incubations and, in *red*, the conditions with HLA-E binding peptides. Graph shows MFI values from the histogram. **b** Myeloma cells were pre-incubated with peptides and controls as described in A for 2 h. Then, IL-2-activated NK cells were added and the cells were co-cultured for an additional 12 h. Percentage of CD107a+ cells was determined for three donors in NKG2A+ KIR− and in NKG2A−KIR+ NK cell subsets. Graphs depict means of duplicate cultures per donor. X axis legend is given in the *lower panel* of the figure

Most of the cell lines in our panel expressed relatively low levels of HLA-E as compared to the levels observed in vivo in myeloma patients and in mice, and therefore, we questioned whether a higher, more clinically relevant level of HLA-E expression would inhibit degranulation of activated NKG2A+ NK cells. HLA-E expression was enhanced on one of the HLA-E^low^ cell lines (U266) by pre-incubation with A1- or B7-peptides. Both peptides are natural binders of HLA-E derived from the leader sequence of HLA-A1 or HLA-B7 allotypes and have been shown to enhance surface HLA-E levels by stabilization of the molecule on the cell surface [12]. Indeed, the incubation with either A1- or B7-peptide enhanced the level of HLA-E on U266 as compared to a non-HLA-E binding control peptide or DMSO (Fig. 6a). Subsequent co-culture experiments with U266- and IL-2-activated allogeneic NK cells revealed that in the control conditions (U266 alone, with DMSO or control peptide), 40–50 % of the NK cells expressed CD107a (Fig. 6b). In response to U266 pre-incubated with A1- or B7-peptide, the percentage of CD107a+ KIR-NKG2A+ NK cells was reduced from 40 to 50 % to only 15–20 %. Degranulation of KIR+NKG2A− NK cells was not influenced by pre-incubation of U266 cells with A1- or B7-peptide. These data support that the upregulation of HLA-E on myeloma cells that are in principle sensitive to NK cell kill, renders these cells resistant to NKG2A+ NK cells, indicating that the selection of NKG2A-negative NK cells is essential to efficiently eliminate HLA-E positive myeloma cells.

## Discussion

Despite the introduction of novel drugs to treat multiple myeloma, this disease has remained largely incurable. NK cells can be novel immunotherapeutic candidates, and their safety has been documented in clinical trials [17]. Exploitation of the full potential of NK cells requires selection of NK cell subsets with the highest possibility of triggering anti-myeloma and identification of the factors important for sustaining NK cell activity in vivo. In our quest of further refining NK cell therapy, we evaluated the clinical relevance of HLA-class I and HLA-E for the anti-myeloma response of alloreactive NK cells.

Expression of HLA-E in primary myeloma has not been reported, and the present study shows that HLA-E is indeed expressed by CD38^high^ cells of myeloma or PCL patients. Our data also demonstrate that primary myeloma cells remain positive for HLA-class I, which has been described before [28–30]. HLA-class I was also strongly expressed by all cell lines in our panel. HLA-E expression, on the other hand, was lower on cell lines than on primary myeloma cells and ranged from completely absent, e.g., on OPM-1, to very low on most of the other myeloma lines. Low levels of HLA-E on solid tumor cell lines were also reported in three previous studies with 30–37 different in vitro cultured cell lines per study mainly of solid tumor origin [42–44]. The most striking observation in our study was that the HLA-E level on U266 cells grown in the BM of RAG-2^−/−^ *γ*c^−/−^mice was considerably higher than the level on in vitro cultured U266 and was relatively comparable to the expression levels in myeloma patients. Thus, there seems to be a difference between in vitro and in vivo expression levels of HLA, which might be the consequence of supportive factors present in vivo that are lacking in vitro. This observation is highly relevant for the interpretation of in vitro NK cell studies using HLA-E^low^ tumor cells in general [45] because the in vitro situation might be very limiting in predicting the actual situation in cancer patients.

In a co-culture system allowing the synchronized analysis of degranulation of NK cell subsets, we identified HLA-E as a potent inhibitory factor for anti-myeloma reactivity of NK cells expressing the inhibitory NKG2A receptor (Fig. 5). The impact of HLA-E was most pronounced in the experiment where blocking of HLA-E enhanced degranulation of KIR-NKG2A+ NK cells and in the experiment where HLA-E binding peptides reduced KIR-NKG2A+ NK cell degranulation via the upregulation of HLA-E. This is consistent with the inhibitory role of HLA-E described in AML and ALL [32]. A limitation of this study is lack of use of anti-HLA-E fragment associated with antigen binding (Fab). Therefore, we cannot fully exclude the possibility that the enhanced degranulation upon HLA-E antibody incubation was caused by triggering of ADCC. However, in our study, HLA-E blocking did not enhance degranulation of KIR+NKG2A− subsets which also express CD16 and can mediate ADCC, suggesting that the enhanced degranulation of KIR-NKG2A+ NK subsets did not trigger ADCC at global NK cell level. Lack of ADCC triggering by the antibody might be explained by the fact that the HLA-E antibody was mouse IgG1 isotype, which has been shown to poorly bind to human Fc receptors on monocytes [46]. Another possible limitation of our study is that we did not confirm our results with MM cells from patient samples due to the very poor survival capacity of primary MM cells in culture. Nevertheless, because we showed that primary MM cells express relatively high levels of HLA-E, it will be important to take the inhibitory effects of HLA-E into account in the design of future (clinical) studies. Because a large fraction of circulating NK cells and the majority of NK cells in clinical products are NKG2A+, HLA-E can be expected to have major impact on overall NK cell alloreactivity against HLA-E-expressing tumors. HLA-E-mediated tumor resistance might at least partly explain the negative clinical trail results obtained so far. Our current data provide a strong argument to use NKG2A-negative NK cells in future clinical trials or develop strategies to abrogate inhibition by HLA-E.

Our findings consequently demonstrated a higher killing potential of KIR–ligand-mismatched NK cells as compared to their matched counterparts. We used NK cells from HLA-C1-, HLA-C2-, HLA-Bw4-positive donors to rule out hyporesponsiveness of non-licensed NK cells, and our data with K562 demonstrated that all subsets had more or less comparable intrinsic capability to kill MM cells. Therefore, it seems unlikely that differences between the subsets were caused by, for example, variation in the level of activating receptors. In addition, we have shown before that activating receptors (e.g., NKG2D and NCRs) are homogenously distributed on CD56+ cells [24]. Previously, bortezomib-induced downregulation of HLA-class I [47], KIR-blockade with IPH2101 [48] and HLA-blockade with W6/32 [16] have been shown to enhance killing of myeloma cells by NK cells. Our data are in agreement with these studies and confirm the relevance of HLA-class I in an alternative experimental setup. An advantage of our system is that it allows the simultaneous analysis of individual subsets, which additionally revealed that the anti-myeloma advantage of mismatched NK cells was reduced by co-expression of one or more matched KIR. Hence, selection of NK cell subsets with exclusively ligand-mismatched KIRs will be important to enhance clinical efficacy.

We recently showed that IL-2 activation is required for NK cell anti-myeloma responses in clinically relevant hypoxic conditions [24]. In addition, we showed in that study that expression of HLA-class I, HLA-E and the NKG2D ligands MICA/B and ULPB1-2 by MM cells was not influenced by hypoxia [24]. Here, we demonstrate that, even though IL-2 activation enhanced degranulation of all NK cell subsets, KIR–ligand-mismatched NK cells still mediated a more potent anti-myeloma response than matched NK cells. Importantly, we did not observe a difference between anti-myeloma responses at 21 and 0.6 % O_2_, indicating that for all subsets, activation was sufficient to overcome the inhibitory effect of hypoxia. Recently, pre-activation of NK cells with a combination of IL-12/IL-15/IL-18 proved to be more efficient in enhancing NK cell effector function as compared to activation with IL-2 or IL-15 alone [49, 50]. Thus, cytokines other than IL-2 might enhance the therapeutic potential of alloreactive NK cells even further.

We acknowledge that in our system, HLA-class I and HLA-E were not the only factors determining the magnitude of the anti-myeloma response, i.e., HLA-E blocking did not completely restore degranulation, and in response to some cell lines (e.g., UM-9), even fully KIR–ligand-mismatched NK cells did not vigorously degranulate (Fig. 4a). Previously, the activating receptors NKG2D, NCRs and DNAM-1 have shown to be important for NK cell anti-myeloma activity [28, 29, 51]. Although our study was not designed to investigate the role of activating ligands, analysis of the NKG2D ligands MICA/B and ULPB2 showed that most myeloma cell lines in our panel expressed at least one of these ligands (supplemental table S2). UM-9, however, expressed very low levels of all three ligands, which might explain the low capacity to activate NK cells. To develop effective immunotherapeutic strategies for resistant myeloma, synergy between alloreactive NK cells and drugs such as lenalidomide and 17-AAG should be evaluated because these drugs have been shown to sensitize myeloma by enhancing the expression of activating ligands [48, 52].

In summary, our results show that KIR–HLA-class I and NKG2A–HLA-E interactions are highly relevant for NK cell reactivity against myeloma. For accurate prediction of in vitro data to patient’s reality, two relevant in vivo realities have to be taken into account: (1) In the bone marrow myeloma, cells reside under hypoxic conditions and (2) in vivo, myeloma cells express both HLA-class I and HLA-E. Infusion of a high number of cytokine-activated alloreactive NKG2A-negative, KIR–ligand-mismatched NK cells or the use of KIR-blocking antibody (IPH2101) may help to improve the efficacy of alloreactive NK cell therapy.

### Electronic supplementary material

Below is the link to the electronic supplementary material.

Supplementary material 1 (PDF 377 kb)

## Abbreviations

ADCC: Antibody-dependent cellular cytotoxicity
AML: Acute myeloid leukemia
BM: Bone marrow
Fab: Fragment antigen binding
GFP: Green fluorescence protein
GvHD: Graft-versus-host disease
HLA: Human leukocyte antigen
HSCT: Hematopoietic stem cell transplantation
KIR: Killer immunoglobulin-like receptor
MFI: Mean fluorescence intensity
MM: Multiple myeloma
NK: Natural killer
PCL: Plasma cell leukemia
