# Supplementary material for: Optimal selection of natural killer cells to kill myeloma: the role of HLA-E and NKG2A
Source: Cancer Immunol Immunother. 2015 Apr 29;64(8):951–63. doi: 10.1007/s00262-015-1694-4 (PMC4506464; doi:10.1007/s00262-015-1694-4)
Supplement: Supplementary file 1 — Supplementary material 1 (PDF 377 kb) [file 262_2015_1694_MOESM1_ESM.pdf]

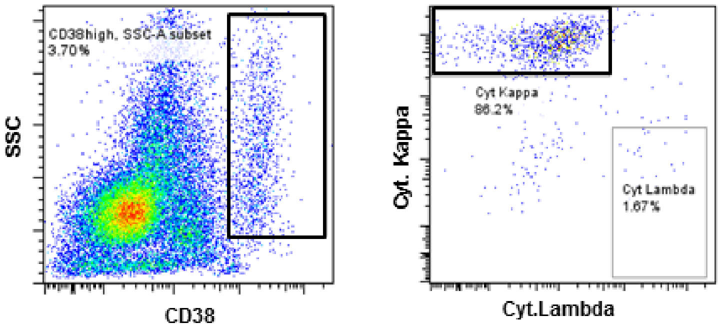

Supplemental figure S1: Patient derived primary CD38^{high} myeloma cells display skewed expression of intracellular kappa- or lambda-light chain. Cells obtained from bone marrow aspirates of patients with myeloma (n=8) or plasma cell leukemia (PCL; n=1) were stained for surface CD38 and for intracellular kappa- and lambda-light chains and were analyzed by flowcytometry. Dotplots show representative data from one myeloma patient. Expression of intracellular kappa- and lambda-light chains is depicted for the CD38^{high} subset.

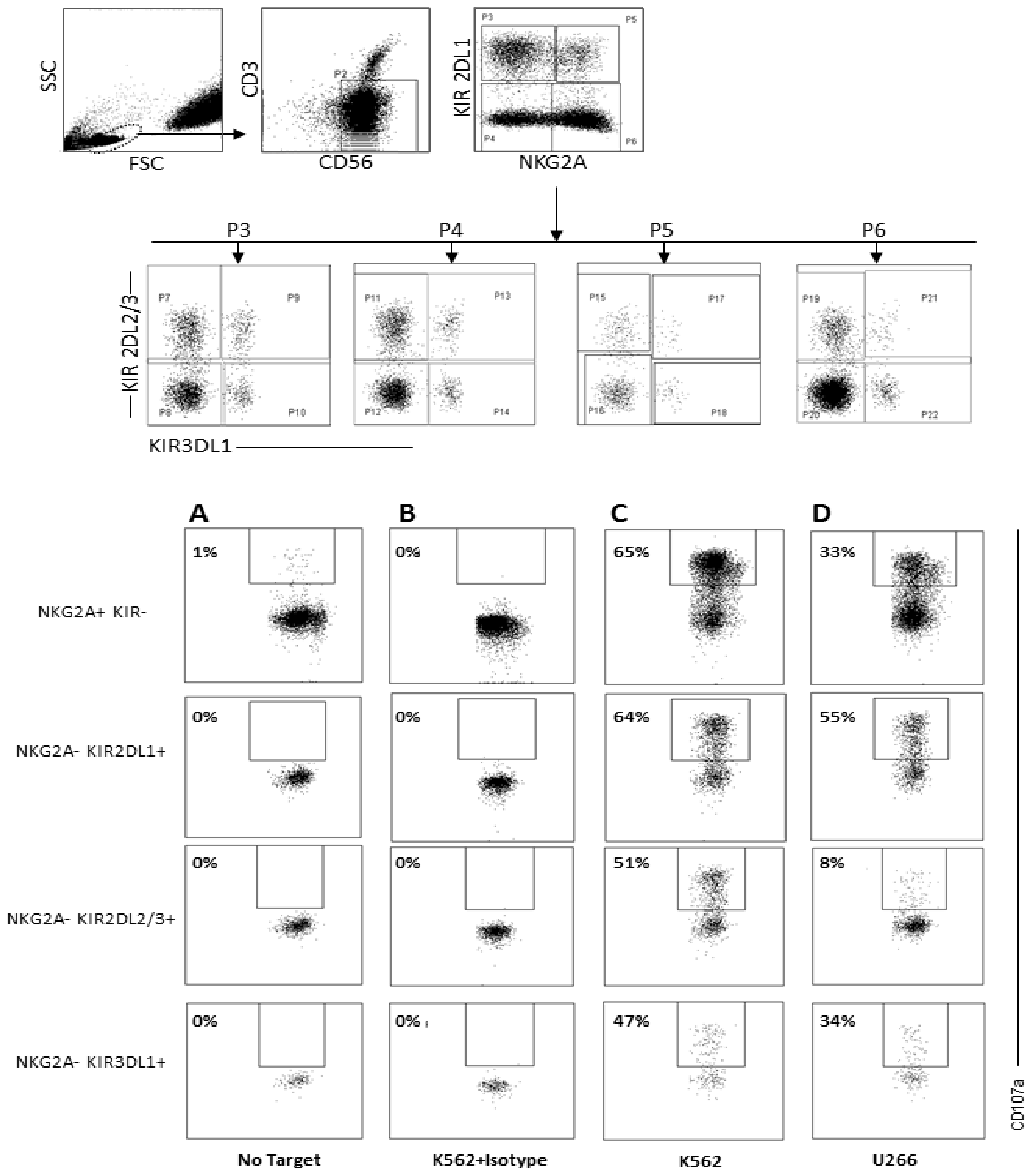

Supplemental figure S2: Gating strategy for CD107a degranulation assay. Myeloma cell lines or K562 were co-cultured with NK cells isolated from peripheral blood in the presence of anti-CD107a (row A,C,D) or an isotype control (row B) at 21% of oxygen. After 12 hours, cells were stained for KIR and NKG2A, and degranulation (CD107a+) was measured by flow cytometry. For analysis, NK cells (CD3-CD56+) were subdivided into 16 subpopulations based on their expression of different inhibitory receptors. The percentage of CD107a+ cells in each of the 16 subsets was analyzed. Dotplots show data for the subsets expressing only one of the inhibitory receptors; i.e. single positive for NKG2A, KIR2DL1, KIR2DL2/3 or KIR3DL1 upon co-culture with U266 or the positive control cell line K562.

Supplemental table S1: Matched and mismatched KIRs based on genotypic expression of HLA epitopes

Cell line	HLA genotype	Matched KIR	Mismatched KIR
**U266**	C1+ C2- Bw4-	KIR2DL2/3	KIR2DL1, KIR3DL1
**L363**	C1+ C2- Bw4-	KIR2DL2/3	KIR2DL1, KIR3DL1
**LME-1**	C1+ C2- Bw4-	KIR2DL2/3	KIR2DL1, KIR3DL1
**UM-9**	C1+ C2- Bw4-	KIR2DL2/3	KIR2DL1, KIR3DL1
**RPMI-8226/s**	C1+ C2+ Bw4-	KIR2DL1, KIR2DL2/3	KIR3DL1
**OPM-1**	C1+ C2+ Bw4-	KIR2DL1, KIR2DL2/3	KIR3DL1
**XG-1**	C1+ C2+ Bw4+	KIR2DL1, KIR2DL2/3, KIR3DL1	No mismatch possible

Matched and mismatched KIRs for each cell line were classified based on the genotypic expression of HLA-class I epitopes as determined by luminex-SSO.

Table S2: Expression of activating NK cell ligands by myeloma cell lines

Isotype Control MFI

Cell line	MICA	MICB	ULBP2
**U266**	958	401	794
**L363**	1061	558	829
**LME-1**	826	429	863
**UM-9**	1017	511	346
**RPMI-8226/s**	1127	440	644
**OPM-1**	913	341	1051
**XG-1**	699	294	687
**K562**	1009	456	957

Positive stain MFI

Cell line	MICA	MICB	ULBP2
**U266**	2414	1305	783
**L363**	1636	1080	1069
**LME-1**	549	867	946
**UM-9**	719	972	450
**RPMI-8226/s**	2311	1007	912
**OPM-1**	917	1214	2714
**XG-1**	725	633	815
**K562**	1383	1692	1359

Normalized MFI

Cell line	MICA	MICB	ULBP2
**U266**	2.5	3.3	1.0
**L363**	1.5	1.9	1.3
**LME-1**	0.7	2.0	1.1
**UM-9**	0.7	1.9	1.3
**RPMI-8226/s**	2.1	2.3	1.4
**OPM-1**	1.0	3.6	2.6
**XG-1**	1.0	2.2	1.2
**K562**	1.4	3.7	1.4

Myeloma cell lines were stained for activating NKG2D ligands and expression was analyzed by flowcytometry. Table depicts MFI of the isotype, Positive staining MFI of the activating ligand and relative expression (=MFI of the activating ligand divided by the MFI of the isotype of the same cell lines). Data are representative of two-three experiments.
